# Ferrocene-Based Bioactive Bimetallic Thiourea Complexes: Synthesis and Spectroscopic Studies

**DOI:** 10.1155/2015/386587

**Published:** 2015-10-08

**Authors:** Shafqat Ali, Ghulam Yasin, Zareen Zuhra, Zhanpeng Wu, Ian S. Butler, Amin Badshah, Imtiaz ud Din

**Affiliations:** ^1^Key Laboratory of Carbon Fiber and Functional Polymers, Beijing University of Chemical Technology Ministry of Education, Beijing 100029, China; ^2^Department of Chemistry, McGill University, Montreal, QC, Canada H3A 2K6; ^3^Department of Chemistry, Quaid-i-Azam University, Islamabad 45320, Pakistan

## Abstract

Bioactive 1,1′-(4,4′-di-ferrocenyl)di-phenyl thiourea and various metal complexes of this ligand have been successfully synthesized and characterized by using physicoanalytical techniques such as FT-IR and multinuclear (^1^H and ^13^C) NMR spectroscopy along with melting point and elemental analyses. The interaction of the synthesized compounds with DNA has been investigated by using cyclic voltammetric and viscometric measurements. The intercalation of the complexes into the double helix structure of DNA is presumably occurring. Viscosity measurements of the complexes have shown that there is a change in length and this is regarded as the least ambiguous and the most critical test of the binding model in solution. The relative potential of the complexes as anti-bacterial, antifungal, and inhibition agents against the enzyme, alkaline phosphatase EC 3.1.3.1, has also been assessed and the complexes were found to be active inhibitors.

## 1. Introduction

One of the essential goals in medical community is to introduce the new anticancer and antimicrobial therapeutic agents. Cancer treatment using metal-based drugs is one of the very effective strategies; for example, platinum drugs cisplatin, carboplatin, and oxaliplatin are routinely used in the clinic to kill cancerous cells but their use has also been limited due to inherent and acquired resistance and the presence of a number of dose-limiting side effects [1, 2]. The search for better metal-based drugs having the ability to overcome problems of drug resistance and side effects associated with platinum based chemotherapy constitutes the foundation of bioorganometallic chemistry. Ferrocene and its derivatives have played an important role as potential chemotherapeutics in particular; considerable attention has been paid to their antitumour, anti-inflammatory, antimicrobial, cytotoxic, and DNA cleaving agents with respect to cancer cells [1–4]. The stability, electroactivity, and high spectroscopic activity of ferrocene-based organometallics make them promising candidates for many biological applications [5]. The presence of the ferrocenyl moiety enhances activity due to its reversible redox behaviour and increases cell permeability due to its lipophilic nature [6]. It has been reported that when ferrocene was incorporated into tamoxifen, the anticancer activity of the drug is enhanced [7]. Ferrocene derivatives may bind to the DNA via both covalent and noncovalent modes of interaction. The anticancer activity of ferrocene derivatives is found to be dependent on the oxidation state of iron in the ferrocene moiety with some results indicating that the Fe(II) ferrocenyl compound is more active than Fe(III) ones [8]. The results of the study on ferrocifen as one of the Fe(II) compounds indicate that the ferrocifens act by changing the conformation of the receptor protein [9]. Binding of ferrocifen to ER*β* is thought to lead to its dimerization followed by attachment of the dimerized species to a particular region of DNA. The electron transfer reaction involving the ferrocenium ion in vivo or the ferrocifen-ER*β* complex may generate reactive oxygen species (ROS) such as hydroxyl radicals (^∙^OH). ROS produced can cause damage to DNA [10] and may also be responsible for anticancer activity through the formation of radical metabolites that bring about biological damage in the cancer cell [11, 12].

Many researchers have reported that thiourea-based complexes show effective results against various biological activities due to the presence of thiocarbonyl moiety which affect biochemical action by the lipophilicity/hydrophilicity and electronic properties of the compounds [13–16]. Herein we report the synthesis, characterization, and investigation of DNA interaction, enzymatic studies, and antibacterial and antifungal activities of various metal complexes of ferrocene-based thiourea and we believe that this study will provide useful information on various biological domains and thus will be very helpful to the design of new drug.

## 2. Materials and Methods

### 2.1. Chemicals and Instrumentation

Ferrocene, hydrochloric acid, sodium nitrite, acetonitrile, dimethyl sulfoxide (DMSO), ethanol, diethyl ether, carbon disulfide, triethylamine, metal salts (Pd, Ag, Cd, Zn, Hg, etc.), ammonium formate, zinc-dust, and alkaline phosphatase (ALP, EC 3.1.3.1) were obtained from E. Merck and Aldrich (Pakistan). All solvents were dried and purified before use according to the reported methods [18]. Elemental analyses (CHNS) were performed using an in-house instrument, Leco CHNS-932 Elemental Analyzer. Melting points were measured using a BIO COTE Model SMP10 melting point apparatus. The FT-IR spectra (4,000–400) cm^−1^ were obtained using KBr disks on a Thermo Scientific Nicolet-6700 FT-IR spectrometer. The NMR spectra of the complexes were recorded using a Bruker Avance 300 MHz NMR spectrometer.

### 2.2. Synthesis of 1,1′-(4,4′-Di-ferrocenyl)di-phenyl Thiourea (**Ft**)

3-Ferrocenylaniline was synthesized in accordance with the methodology reported earlier [17]. An ethanolic solution of 3-ferrocenylaniline (2.0 mmol) was added dropwise to a solution of carbon disulfide (1.0 mmol) containing a few drops of triethylamine in an ice bath (0–5°C) and then reaction mixture was stirred overnight at room temperature. The progress of the reaction was monitored by TLC. After completion, the reaction mixture was filtered off and the residue was recrystallized from acetonitrile to obtain the symmetrical ferrocene-based thiourea ligand (**Ft**); Yield 65%, m.p., 180°C. Molecular formula (Mol. wt.) is found as C_33_H_24_Fe_2_N_2_S (596). FT-IR (*ν*, cm^−1^): Fe-Cp (490 cm^−1^), NH (3354 cm^−1^), sp^2^ CH (3084 cm^−1^), C=C Ar (1587 cm^−1^), meta-disubstituted benzene (883 cm^−1^), C=S (740 cm^−1^). ^1^H NMR (300 MHz, CDCl_3_): *δ* 4.09 (s, 5H, C_5_H_5_), 4.36 (s, 2H, C_5_H_4_), 4.66 (s, 2H, C_5_H_4_), 7.59 (s, 1H, C_6_H_4_), 7.22 (d, 1H, *J* = 7.8 Hz, C_6_H_4_), 7.42 (t, 1H, *J* = 7.8 Hz, C_6_H_4_), 7.34 (t, 1H, *J* = 7.8 Hz, C_6_H_4_), 7.99 (s, 1H, NH) ppm.

^13^C NMR (75 MHz, CDCl_3_): *δ* 69.75, 69.35, 66.67, 83.99, 137.15, 122.78, 141.54, 124.72, 129.53, 122.33, 179.77 ppm. Elemental analysis Cal.(%): C, 66.46; H, 4.73; N, 4.70; S, 5.38. Found (%): C, 66.39; H, 4.74; N, 4.68; S, 5.34.

### 2.3. Synthesis of Metal Complexes

The target compounds (**1–5**) were synthesized by the following general procedure.

An acetonitrile solution of ferrocene-based thiourea (**Ft**) was added dropwise to an acetonitrile solution of the appropriate metal salt (Zn, Cd, Hg, Pd, and Ag) in a 1 : 2 mole ratio and the reaction mixture was stirred for 4–6 h at room temperature; the extent of the reaction was monitored by TLC. After completion of the reaction, the mixture was filtered off and the residue was isolated. This solid material was dissolved in dichloromethane and then recrystallized using *n*-hexane: chloromethane mixture (1 : 3). Unfortunately, the crystals obtained were not of sufficient quality for single-crystal X-ray diffraction analysis.

### 2.4. FT-IR and Multinuclear (^1^H and ^13^C) NMR Studies

The FT-IR and multinuclear (^1^H and ^13^C) NMR spectral data for the complexes are as follows.

For compound** 1** with molecular formula (Mol. wt.) found as C_66_H_56_Cl_2_Fe_4_N_4_S_2_Zn (1328), FT-IR (*ν*, cm^−1^): Fe-Cp (486 cm^−1^), NH (3204 cm^−1^), sp^2^ CH (2962 cm^−1^), C=C Ar (1525 cm^−1^), meta-disubstituted benzene (880 cm^−1^), C=S (724 cm^−1^). ^1^H NMR (300 MHz, DMSO): *δ* 4.06 (s, 10H, C_5_H_5_), 4.34 (s, 4H, C_5_H_4_), 4.73 (s, 4H, C_5_H_4_), 7.71 (s, 2H, C_6_H_4_), 7.27 (d, 2H, *J* = 7.5 Hz, C_6_H_4_), 7.33 (t, 2H, *J* = 7.5 Hz, C_6_H_4_), 7.36 (d, 2H, *J* = 7.5 Hz, C_6_H_4_), 9.81 (s, 2H, NH) ppm. ^13^C NMR (75 MHz, DMSO): *δ* 69.85, 69.35, 66.56, 85.18, 139.85, 121.94, 139.88, 122.70, 128.82, 121.73, 177.18 ppm. Elemental analysis Cal.(%): C, 59.65; H, 4.25; N, 4.22; S, 4.83; Found (%): C, 59.71; H, 4.21; N, 4.19; S, 4.83. Yield 45% and m.p., 240°C.

For compound** 2** with molecular formula (Mol. wt.) C_66_H_56_Cl_2_Fe_4_N_4_S_2_Cd (1376), FT-IR (*ν*, cm^−1^): Fe-Cp (484 cm^−1^), NH (3290 cm^−1^), sp^2^ CH (3097 cm^−1^), C=C Ar (1590 cm^−1^), meta-disubstituted benzene (885 cm^−1^), C=S (728 cm^−1^). ^1^H NMR (300 MHz, DMSO): *δ* 4.09 (s, 10H, C_5_H_5_), 4.35 (s, 4H, C_5_H_4_), 4.66 (s, 4H, C_5_H_4_), 7.60 (s, 2H, C_6_H_4_), 7.22 (d, 2H, *J* = 7.5 Hz, C_6_H_4_), 7.34 (t, 2H, *J* = 7.8 Hz, C_6_H_4_), 7.41 (d, 2H, *J* = 7.5 Hz, C_6_H_4_), 10.08 (s, 2H, NH) ppm. ^13^C NMR (75 MHz, DMSO): *δ* 69.85, 68.50, 65.75, 83.05, 136.20, 121.72, 140.51, 121.26, 128.59, 123.65, 175.56 ppm. Elemental analysis Cal.(%): C, 57.61; H, 4.10; N, 4.07; S, 4.66; Found (%): C, 57.44; H, 4.14; N, 4.04; S, 4.65. Yield 60% and m.p., 230°C.

For compound** 3** with molecular formula (Mol. wt.) calculated as C_68_H_56_Cl_2_Fe_4_HgN_6_S_2_ (1464), FT-IR (*ν*, cm^−1^): Fe-Cp (490 cm^−1^), NH (3091 cm^−1^), sp^2^ CH (2928 cm^−1^), C=C Ar (1576 cm^−1^), meta-disubstituted benzene (893 cm^−1^), CN (2353 cm^−1^), C=S (631 cm^−1^). ^1^H NMR (300 MHz, DMSO): *δ* 4.22 (s, 10H, C_5_H_5_), 4.29 (s, 4H, C_5_H_4_), 4.62 (s, 4H, C_5_H_4_), 6.77 (s, 2H, C_6_H_4_), 6.44 (d, 2H, *J* = 7.2 Hz, C_6_H_4_), 7.11 (t, 2H, *J* = 7.8 Hz, C_6_H_4_), 7.21 (d, 2H, *J* = 7.5 Hz, C_6_H_4_), 10.16 (s, 2H, NH) ppm. ^13^C NMR (75 MHz, DMSO): *δ* 68.85, 68.60, 65.85, 83.15, 136.31, 121.85, 140.62, 121.39, 128.60, 123.76, 176.65, 144.62 ppm. Elemental analysis Cal.(%): C, 54.14; H, 3.86; N, 5.83; S, 4.38; Found (%): C, 54.11; H, 3.89; N, 5.83; S, 4.42. Yield 70% and m.p., 240°C.

For compound** 4** with molecular formula (Mol. wt.) found as C_66_H_56_Cl_2_Fe_4_N_4_PdS_2_ (1370), FT-IR (*ν*, cm^−1^): Fe-Cp (483 cm^−1^), NH (3200 cm^−1^), sp^2^ CH (3090 cm^−1^), C=C Ar (1584 cm^−1^), meta-disubstituted benzene (878 cm^−1^), C=S (734 cm^−1^). ^1^H NMR (300 MHz, DMSO): *δ* 4.06 (s, 10H, C_5_H_5_), 4.35 (s, 4H, C_5_H_4_), 4.73 (s, 4H, C_5_H_4_), 7.71 (s, 2H, C_6_H_4_), 7.25 (d, 2H, *J* = 7.8 Hz, C_6_H_4_), 7.32 (t, 2H, *J* = 6.9 Hz, C_6_H_4_), 7.39 (d, 2H, *J* = 6.9 Hz, C_6_H_4_), 9.81 (s, 2H, NH) ppm. ^13^CNMR (75 MHz, DMSO): *δ* 68.75, 68.50, 65.75, 83.05, 136.21, 121.75, 140.52, 121.29, 128.50, 123.66, 171.45 ppm. Elemental analysis Cal.(%): C, 57.86; H, 4.12; N, 4.09; S, 4.68; Found (%): C, 57.83; H, 4.15; N, 4.07; S, 4.69. Yield 60% and m.p., 210°C.

For compound** 5** with molecular formula (Mol. wt.) C_66_H_56_Fe_4_N_5_O_3_S_2_Ag (1361), FT-IR (*ν*, cm^−1^): Fe-Cp (483 cm^−1^), NH (3290 cm^−1^), sp^2^ CH (3083 cm^−1^), C=C Ar (1583 cm^−1^), meta-disubstituted benzene (881 cm^−1^), NO-asym (1580 cm^−1^), NO-sym (1541 cm^−1^), C=S (724 cm^−1^). ^1^H NMR (300 MHz, DMSO): *δ* 4.03 (s, 10H, C_5_H_5_), 4.36 (s, 4H, C_5_H_4_), 4.77 (s, 4H, C_5_H_4_), 7.49 (s, 2H, C_6_H_4_), 7.16 (d, 2H, *J* = 6.9 Hz, C_6_H_4_), 7.34 (t, 2H, *J* = 7.5 Hz, C_6_H_4_), 7.42 (d, 2H, *J* = 7.5 Hz, C_6_H_4_), 10.22 (s, 2H, NH) ppm. ^13^CNMR (75 MHz, DMSO): *δ* 69.89, 69.28, 66.91, 84.73, 138.98, 123.82, 140.44, 122.7, 129.53, 116.38, 172.43 ppm. Elemental analysis Cal.(%): C, 58.18; H, 4.11; N, 5.14; S, 4.71; Found (%): C, 58.19; H, 4.13; N, 5.19; S, 4.74. Yield 50% and m.p., 230°C.

### 2.5. DNA Binding Studies by Cyclic Voltammetry and Viscometry

Cyclic voltammetric (CV) measurements were performed in a single compartment cell with a three-electrode configuration using an Eco Chemie Auto lab PGSTAT 12 potentiostat/galvanostat (Utrecht, The Netherlands) instrument equipped with the electrochemical software package GPES 4.9. The three-electrode system consisted of reference electrode: RE-1B silver-silver chloride (Ag/AgCl) saturated with sodium chloride (NaCl) of length 70 mm and outer diameter of 6.0 mm (ALS category number 012167), a Beckman platinum wire of thickness 0.5 mm with an exposed end of 10 mm as the counter electrode, and a bare glassy carbon electrode (surface area of 0.071 cm^2^) as working electrode. The voltammogram of a known volume of the test solution was recorded in the absence of calf thymus DNA (CT-DNA) after flushing out oxygen by purging with argon gas for 10 min just prior to each experiment. The procedure was then repeated for systems with constant concentration of the compounds** Ft** and** 1**–**5** (1 mM) and increasing concentration of CT-DNA (1 mL of 20, 40 *μ*M). All the sample solutions were prepared in 20% aqueous DMSO and buffered at pH 7 by phosphate buffer (0.1 M NaH_2_PO_4_ + 0.1 M NaOH); 0.1 mM potassium chloride (KCl) was used as supporting electrolyte. The working electrode was cleaned after every electrochemical assay [19]. The stock solution of CT-DNA (200 *μ*M) was prepared by using doubly distilled water and stored at 4°C. The concentration of CT-DNA was determined by UV absorbance at 260 nm (molar coefficient Є of CT-DNA was taken as 6600 M^−1 ^cm^−1^). The nucleotide to protein (N/P) ratio of 1.85 was obtained from the ratio of absorbance at 260 and 280 nm (A260/A280 = 1.85), providing evidence for protein-free DNA [20].

Viscosity measurements were carried out using Oswald Viscometer, maintained at a constant temperature at 25.0 ± 0.1°C in a thermostatic bath. A series of solutions were made with varying concentration of DNA and constant concentration of the compound. Flow times were measured with a digital stopwatch, and each solution of the complexes was measured three times, and an average flow time was calculated. Data are presented as *η*/*η*_0_ versus binding ratio [compound]/[DNA], where *η* is the viscosity of DNA in the presence of complex and *η*_0_ is the viscosity of DNA alone. All the experiments were conducted in 0.1 M phosphate buffer (pH 7) at 25°C and the results were the average of three experimental measurements.

### 2.6. Enzyme Inhibition Studies

The basic principle of this study is that the alkaline phosphatase in the sample catalyzes the hydrolysis of colorless p-nitrophenyl phosphate (p-NPP) to give p-nitrophenol and inorganic phosphate. At the pH of the assay (alkaline), the p-nitrophenol is in the yellow phenoxide form. The rate of absorbance increase at 405 nm is directly proportional to the alkaline phosphatase activity in the sample. Synthesized compounds** Ft** and** 1**–**5** were screened for their inhibitory activity against the enzyme alkaline phosphatase, EC 3.1.3.1. The enzyme activity was monitored spectrophotometrically at constant temperature (25°C) through the increase in absorbance at 405 nm, which is associated with the hydrolysis of the substrate, para-nitrophenyl phosphate (pNPP). The reaction was started by addition of 40 *μ*L of the enzyme to 2 mL of an assay system in DMSO containing 2 mM pNPP in 0.05 M Na_2_CO_3_-NaHCO_3_ buffer (pH 10.0) at different concentrations of the complexes. Absorption measurements were recorded using a Beckman U-2020 spectrophotometer.

### 2.7. Antibacterial Assay

The successful locally isolated pathogens from (1) urinary tract infections (indigenous uropathogens), that is,* Klebsiella pneumonia* and* Escherichia coli*, and (2) other hospital acquired infections, that is,* Staphylococcus aureus* and* Micrococcus luteus*, were examined for antibacterial activities. All synthesized compounds were tested by a reported method with minor modifications (agar well diffusion assay) [17, 21] where imipenem was used as the standard antibiotic [22]. The whole experiment was performed at pH 7, using appropriate concentration of reagents and McFarland solution as turbidity standard. Using a micropipette, 30 *μ*L of each compound was poured in their respective wells. The incubated time was 24 h at 37°C. The zone of inhibition (%) was calculated for each compound and compared with the standard antibiotic.

### 2.8. Antifungal Assay

Antifungal screening of the synthesized compounds (**Ft** and** 1**–**5**) was carried out against* Aspergillus niger*. Terbinafine was used as standard drug [23]. Different concentrations of each compound (3 mg/mL, 5 mg/mL, and 20 mg/mL) were prepared in 100 mL of DMSO. Tubes were loaded with solutions of each compound, standard drug (negative), and positive control (DMSO) in the growth medium by using a micropipette. Fungal spores were transferred to each growth culture test tube during assay with maintaining pH 4 [24]. These tubes were incubated at human body temperature (37°C) for one week. The same procedure was repeated for 3 times to get better and mean results and it was found that most significant results were obtained for concentration of 20 mg/mL. After required time of incubation, the zones of inhibition were measured and the percentage of fungal inhibition was calculated and compared with the standard drug.

## 3. Results and Discussion

**Scheme 1:**
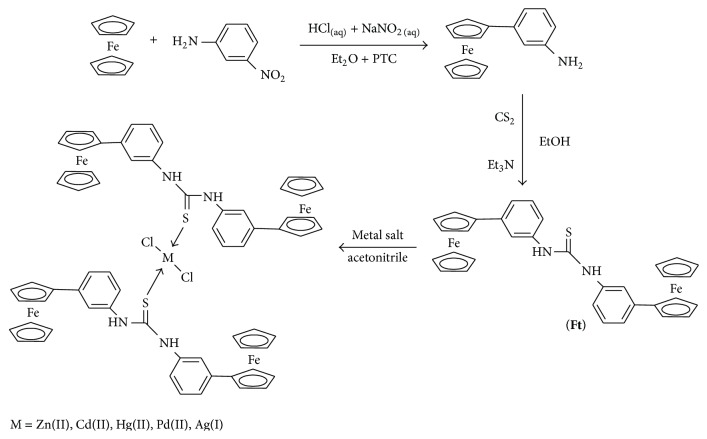
Synthesis of ferrocene-based bimetallic thiourea complexes.

1,1′-(4,4′-Di-ferrocenyl)di-phenyl thiourea was synthesized by the reaction of 3-ferrocenylaniline and carbon disulfide in the presence of triethylamine as a base. Complexes** 1**–**5** were synthesized by mixing the thiourea ligand and different metal salts in a 1 : 2 mole ratio (Scheme 1). Compounds** Ft** and** 1**–**5** are quite stable in moist air. The molecular structures of the synthesized compounds were established on the basis of data obtained by elemental analysis and spectroscopic studies like multinuclear (^1^H and ^13^C) NMR and FT-IR.

### 3.1. Spectroscopic Studies

#### 3.1.1. NMR Spectroscopy

Representative ^1^H NMR data for the compounds are given in the experimental section. The marker peak for** Ft** is the N-H signal that is shifted from 3.70 (3-ferrocenylaniline) to 7.99 proving the formation of the symmetrical ferrocene-based thiourea. A downfield shift in N-H resonance was observed between C-N bonds. The unsubstituted C_5_H_5_ ring of ferrocene appears as a singlet in the ^1^H NMR spectrum at *δ* 4.10, whereas the ortho- and metaprotons on the substituted Cp ring are present at *δ* 4.65 and *δ* 4.33, respectively, which split into three peaks on formation of the compound. One singlet for the five protons of one Cp ring is at *δ* 4.09 ppm and there are two pseudo triplets at *δ* 4.33 and *δ* 4.65 ppm with *J*-values of 6.2 Hz. This splitting of one peak into three peaks provides evidence for the attachment of the substituent of the one Cp ring of the ferrocene. For complexes** 1**–**5**, the N-H signal of the** Ft** became less intense upon coordination and it is shifted downfield from the position in the free ligand. The deshielding is related to an increase of *π*-electron density in the C-N bond upon coordination and it may be due to the development of hydrogen bonding between the H of N-H and the Cl of the metal. The appearance of the N-H signal shows that ligand is coordinated to the metals via sulfur of the** Ft** ligand. A small difference in chemical shift is observed in other hydrogen atoms due to *π*-character. All the protons in the complexes can be identified and the total number of protons estimated from the peak heights of the integration curves agrees well with the expected molecular formulae.

The ^13^C NMR spectral data are also presented in experimental section. The C=S peak appeared at 177.79 ppm and all other peaks within the range confirm the synthesis of** Ft**. For complexes** 1**–**5**, the *δ* (C=S) resonance of the ligand in the complex is shifted upfield by about 2 ppm as compared to the free ligand. The upfield shift is attributed to the lowering of the *δ* (C=S) bond strength producing a partial double bond character in the C-N bond. The shift difference of the C=S resonance may be related to the strength of the metal-sulfur bond. A small deshielding effect is observed for the other carbon atoms, due to an increase in the *π*-character of the C-N bond.

#### 3.1.2. Infrared Spectroscopy

Important IR data for the compounds are presented in experimental section. The characteristic bands were observed: *ν* (C=S) at 740 cm^−1^, *ν* (N-H) for the secondary amine in this case at 3354 cm^−1^, *ν* (meta disubst. benzene) at 883 cm^−1^, *ν* (C-H) aromatic at 3084 cm^−1^, *ν* (C=C) aromatic at 1587 cm^−1^, and *ν* (Fe-Cp) at 490 cm^−1^. These bands indicate the formation of** Ft**. The shift of the bands from those for the initial compound confirms the product formation. For complexes** 1**–**5**, characteristic bands were expected in ranges indicated: *ν* (C=S) around 729–750 cm^−1^, N-H 3204–3220 cm^−1^, and Fc-Cp near 478–486 cm^−1^. There are low frequency shifts in the *ν* (C=S) and *ν* (N-H) bands when compared to those of the free ligand.

### 3.2. DNA Binding Studies through Cyclic Voltammetry

**Figure 1:**
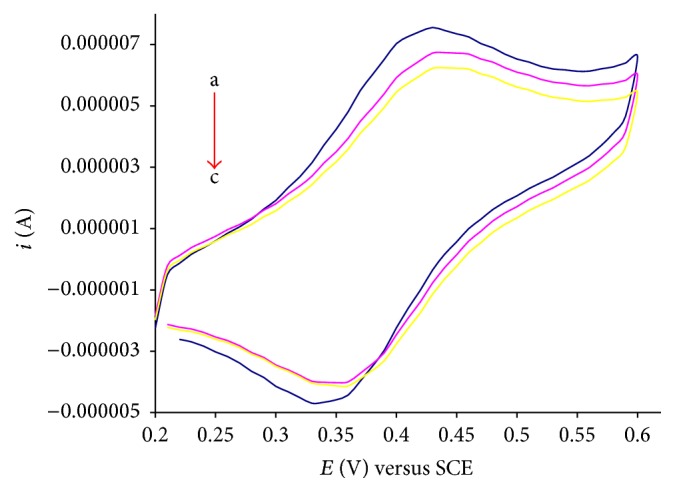
Cyclic voltammograms of 1 mM compound** Ft** recorded at 100 mV/s potential sweep rate on glassy carbon electrode at 25°C in the absence (a) and presence of 1 mL of 20 *μ*M, 40 *μ*M with increasing concentration of CT-DNA (b-c) in 20% aqueous DMSO buffer at pH 7.0; supporting electrolyte 0.1 M KCl.

**Figure 2:**
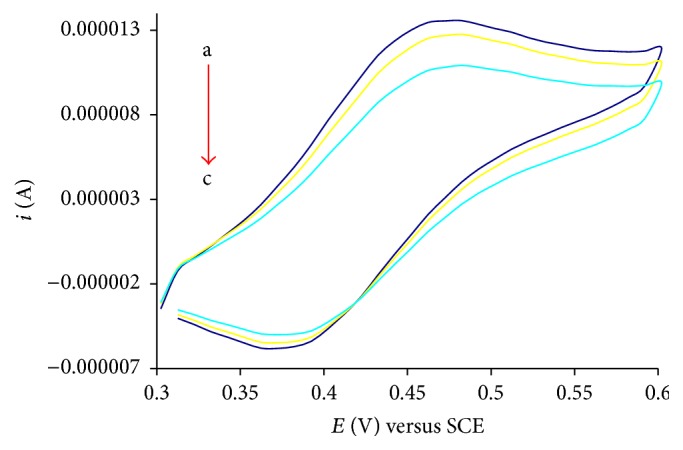
Cyclic voltammograms of 1 mM compound** 1** recorded at 100 mV/s potential sweep rate on glassy carbon electrode at 25°C in the absence (a) and presence of 1 mL of 20 *μ*M, 40 *μ*M with increasing concentration of CT-DNA (b-c) in 20% aqueous DMSO buffer at pH 7.0; supporting electrolyte 0.1 M KCl.

**Figure 3:**
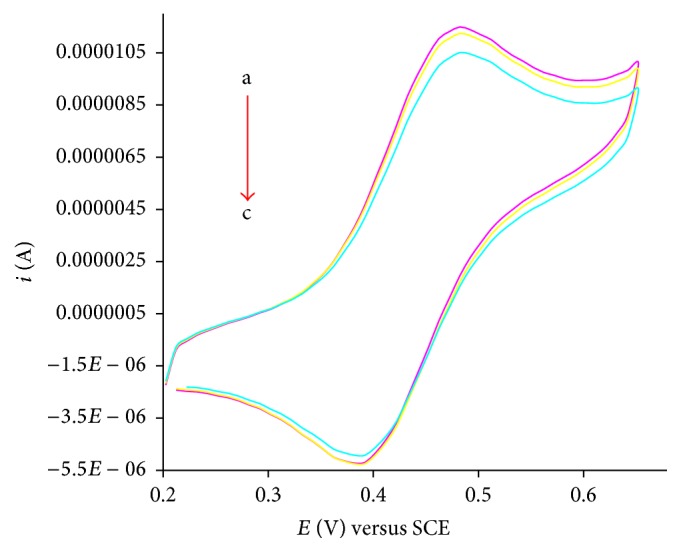
Cyclic voltammograms of 1 mM compound** 4** recorded at 100 mV/s potential sweep rate on glassy carbon electrode at 25°C in the absence (a) and presence of 1 mL of 20 *μ*M, 40 *μ*M with increasing concentration of CT-DNA (b-c) in 20% aqueous DMSO buffer at pH 7.0; supporting electrolyte 0.1 M KCl.

Investigations of drug-DNA interactions have great importance in life science [25]. Interest in understanding the association of drug molecules with duplex DNA has been developed in the hope of understanding the mode of binding [26]. The noncovalent interactions of a drug with DNA may involve three possible modes of interaction: intercalation, groove binding, and electrostatic interactions [27]. There are different techniques which can be used to demonstrate the mode of interaction and the DNA binding parameters. One of the most sophisticated and sensitive techniques is cyclic voltammetry. Voltammetric measurements were performed in a single compartment cell with a three-electrode configuration with the objective of understanding the redox behavior and the DNA binding affinities of** Ft**,** 1**, and** 4** [28–30]. The measurements were carried out with increasing concentration of calf thymus DNA (1 mL of 20 *μ*M, 40 *μ*M) against constant concentration (1 mM) of** Ft**,** 1,** and** 4**. The voltammogram was recorded in the absence and presence of CT-DNA in sample solutions. On addition of increasing concentration of CT-DNA into a 1 mM solution of** Ft**,** 1,** and** 4**, a drop in current *i*_pa_ and a shift in anodic potential are observed (as shown in Figures 1, 2, and 3).

The shift in peak potential is used to investigate mode of interaction between** Ft**,** 1,** and** 4** and DNA. The slightly positive shift in the peak potential is indicative intercalation of the compounds into double helical structure of DNA. The binding ratio of reduced and oxidized species is calculated according to the following equation [19, 31]:

(1) $$\begin{array}{c} {Eb}^{{}^{\circ}}-{Ef}^{{}^{\circ}}=0.05916\log\left(\;\frac{K_{red.}}{K_{oxd.}}\;\right), \end{array}$$

where Eb° and Ef° are the formal potentials of the free and bound forms of drug, respectively. The positive shift indicates intercalation with DNA for** Ft**,** 1,** and** 4**. The drop in current is attributed to diffusion of the drug into the double helical DNA resulting in the formation of a supramolecular complex. As the supramolecular complex is formed, the number of electrons transferred is decreased and hence the drop off in current. The increase in molecular weight of the compound (due to adduct formation with DNA) also justifies the idea that heavy molecules migrate slowly to the electrode and so a decrease in current is observed. The binding constant is determined using the following equation [32]:

(2) $$\begin{array}{c} \frac{1}{\left[\text{DNA}\right]}=\frac{K\left(1-A\right)}{1-\left(i/i_{o}\right)}-K, \end{array}$$

where *K* is the binding constant, *i* and *i*_*o*_ are the peak currents with and without CT-DNA, and *A* is the proportionality constant. The plot of 1/[DNA] versus 1/(1 − *i*/*i*_*o*_) yields binding constants and is listed in Table 1.

The DNA binding affinity of 3-ferrocenylaniline has already been reported by our research group [17]. The DNA binding affinity of 3-ferrocenylaniline is greater than that of** Ft**,** 1,** or** 4**. This difference may be attributed to the mixture of binding modes; that is, the ferrocenyl moiety binds electrostatically to the negatively charged phosphate of the DNA backbone and there is intercalation of the planar phenyl moiety into the base pair pockets. The binding constants of** 1**–**4** and 3-ferrocenylaniline are listed in Table 1.

The free binding energy is calculated from the equation −Δ*G* = RTln⁡*K*. The negative value of free binding energy of** 1**–**4** and 3-ferrocenylaniline in kJ/mol at 25°C shows the spontaneity of compound-DNA interaction [17], as listed in Table 1, while compound** 5** showed almost same behavior as compound** 3**.

### 3.3. DNA Binding Studies through Viscometry

**Figure 4:**
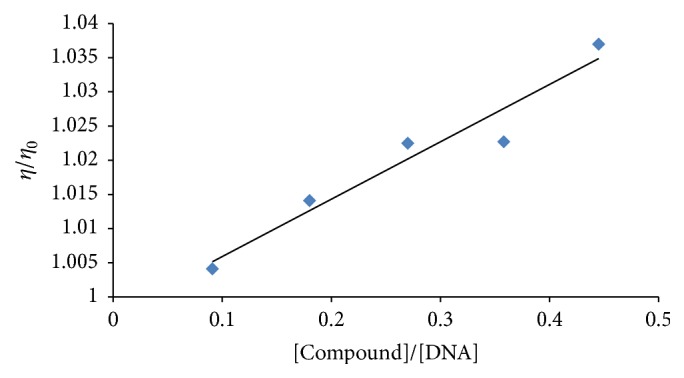
Effect of increasing concentration of compound** 1** on the relative viscosity of DNA at 25°C. [DNA] = 30 *μ*M and [compound-1] = 5–25 *μ*M.

**Figure 5:**
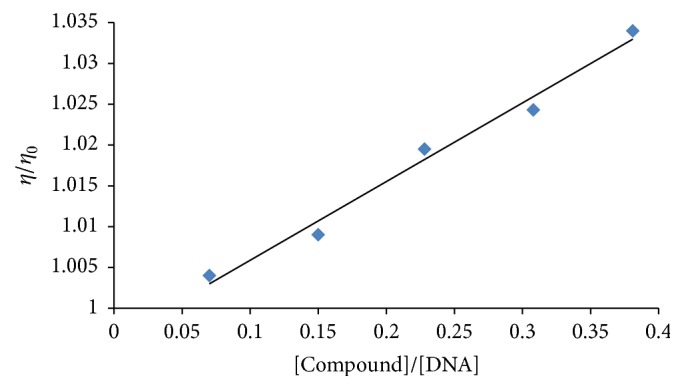
Effect of increasing concentration of compound** 5** on the relative viscosity of DNA at 25°C. [DNA] = 30 *μ*M and [compound-5] = 5–25 *μ*M.

Another useful technique to prove intercalation is the viscosity measurement, which is sensitive to the length change of DNA due to the lengthening of DNA helix as the base pair pockets are widened to accommodate the binding molecule. This technique is regarded as the least ambiguous and the most critical test of the binding mode in solution under appropriate conditions (constant temperature at 25.0 ± 0.1°C in a thermostatic bath). The plots reveal negative changes in (*η*/*η*_0_) with increasing concentration of all compounds. The graph between relative specific viscosity (*η*/*η*_0_) and [compound]/[DNA] for** 1** and** 5** is shown as representation in Figures 4 and 5. This mode of action is suggestive of intercalation that may cause lengthening of the DNA chain [33].

### 3.4. In Vitro Inhibition Studies of Alkaline Phosphatase

**Figure 6:**
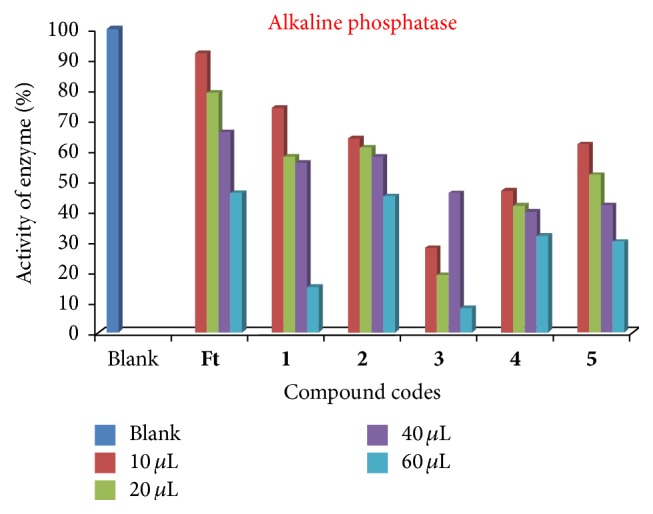
Enzymatic studies (alkaline phosphatase) of compounds** Ft** and** 1**–**5**.

The effect of various concentrations of compounds** Ft** and** 1**–**5** (10 *μ*L, 20 *μ*L, 40 *μ*L, and 60 *μ*L) on the activity of the enzyme, alkaline phosphatase EC 3.1.3.1, was studied for the hydrolysis of p-nitrophenyl phosphate (pNPP). Alkaline phosphatase catalyzes the transfer of phosphate groups to water (hydrolysis) or alcohol (transphosphorylation) using a wide variety of phosphomonoesters and is characterized by high pH optima and a broad substrate specificity [34]. Here, we have practical evidence that the presence of different metals resulted in the deactivation of the enzyme of 40 *μ*L as concentration. The activity of enzyme was markedly decreased by increasing the concentration of the compounds. The % activity of the enzyme (alkaline phosphatase) is presented in Figure 6.

### 3.5. Antibacterial Activity

In vitro evaluation of antibacterial activity was successfully carried out. The experiments were repeated three times and the results are reported as means of at least three determinations and the results are summarized in Table 2. As evident from Table 2,** Ft** and** 1**–**5** exhibited significant inhibitory activity against the two strains,* Staphylococcus aureus* and* Micrococcus luteus*, as compared to standard drug (imipenem) at the tested concentration.

### 3.6. Antifungal Activity

Table 3 summarizes the antifungal activity of the compounds against pathogenic yeast species. The results reveal that all the compounds had promising antifungal activities against* Aspergillus niger* and poor activities against other yeasts. These results suggest that the compound has effective activities against selective yeasts. Iron is essential for microorganisms as a trace nutrient. Moreover, several studies had reported that iron containing organometallic compounds showed good antimicrobial activities [35].

## 4. Conclusion

Ferrocene incorporated bimetallics (**1**–**5**) have been synthesized and successfully characterized. During DNA binding studies, the shift in formal potential reveals the mode of interaction between the complexes and DNA. Compounds** Ft**,** 1,** and** 4** undergo intercalation into the double helix structure of DNA and this result is also supported by viscometric measurements. These complexes have been checked for their alkaline phosphatase activity in the presence and absence of inhibitor which shows that by the addition of inhibitor the activity of enzyme decreases and at higher concentration it is completely inhibited. Compounds** Ft** and** 1–5** are biologically active against Gram-positive bacteria (*S. aureus* and* M. luteus*), Gram-negative bacteria (*E. coli* and* K. pneumoniae*), and selective yeast* A. niger*.

## Figures and Tables

**Table 1:** Binding constant and binding energy values of **1–4** and 3-ferrocenylaniline [17].

Compound	Binding constant (M^−1^)	−Δ*G* (kJ/mol)
**Ft**	3.43 × 10^3^	19.41
**1**	4.63 × 10^3^	20.23
**2**	4.83 × 10^3^	20.41
**3**	4.57 × 10^3^	20.35
**4**	5.85 × 10^3^	20.87
3-Ferrocenylaniline	9.39 × 10^3^	21.67

**Table 2:** Antibacterial activity of **Ft** and **1–5**.

Chemical codes	*Staphylococcus aureus *		*Klebsiella pneumoniae *		*Micrococcus luteus *		*Escherichia coli *	
	(2) (*G* +ve)		(1) (*G* −ve)		(2) (*G* +ve)		(1) (*G* −ve)	
	Radius (mm)	% value	Radius (mm)	% value	Radius (mm)	% value	Radius (mm)	% value
Imipenem	18	100	20	100	18	100	20	100
**Ft**	13	72	02	11	16	89	02	11
**1**	00	00	3	17	3	17	5	28
**2**	3	17	2	11	4	22	3	17
**3**	7	39	3	17	00	00	4	22
**4**	9	50	6	33	7	39	11	61
**5**	3	17	04	20	00	00	03	17

**Table 3:** Antifungal activity of **Ft** and **1–5**.

Compound codes	Concentration (mg/100 mL)	Negative control growth/DMSO (cm)	Culture length (in control) (cm)	Fungal growth length (in sample)	% inhibition of fungal growth
**Ft**	3.00	10.40	11.00	7.10	35.50
	5.00	10.00	11.00	5.60	49.00
	20.00	10.00	11.00	3.30	70.00

**1**	3.00	9.80	10.50	9.70	7.60
	5.00	9.50	10.50	8.60	18.00
	20.00	10.00	10.50	7.00	33.30

**2**	3.00	10.20	11.50	10.20	11.30
	5.00	10.30	11.50	9.50	17.40
	20.00	10.00	11.50	8.10	29.56

**3**	3.00	11.00	12.00	10.50	12.50
	5.00	11.50	12.00	8.20	31.66
	20.00	11.60	12.00	6.30	47.50

**4**	3.00	10.70	10.50	7.30	30.48
	5.00	10.40	10.50	4.50	57.14

**5**	3.00	9.60	10.00	8.40	16.00
	5.00	9.00	10.00	6.30	37.00
	20.00	9.50	10.00	2.80	72.00

Std. drugs = Terbinafine (100%).
